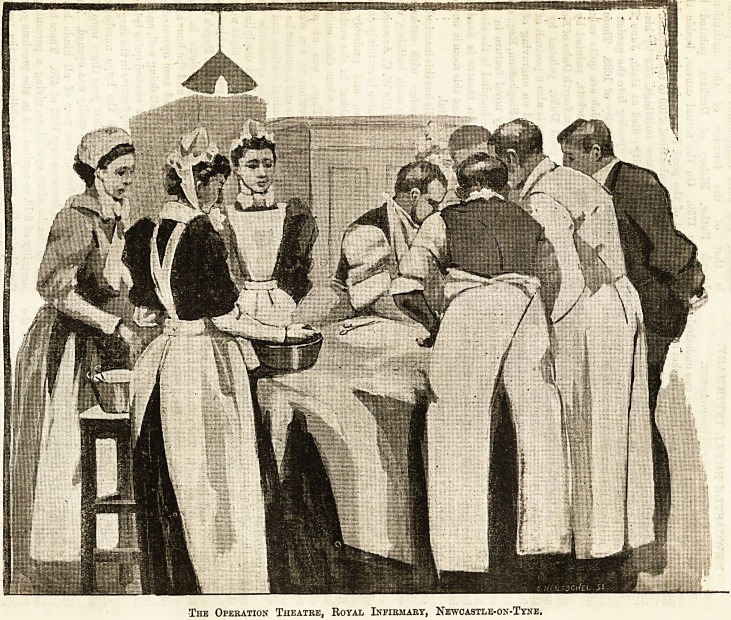# Newcastle Royal Infirmary

**Published:** 1894-03-31

**Authors:** 


### The Institutional Workshop.

### WITHIN THE HOSPITALS.

### NEWCASTLE ROYAL INFIRMARY.

By A Vagrant Correspondent.

The Newcastle Royal Infirmary has two undesirable neighbours front and rear, the cattle market and the Newcastle and Carlisle Railway. All through the year the noise and rattle on the railroad disturbs the patients in the rear, and every week on market days the bustle of buyers and sellers, and the unsymphonic voices of the cattle distract the sensitive patients in the front of the hospital.

Somewhat dark and prison-like is the old exterior of the infirmary, and its architecture would be altogether uninteresting but for the two Queen Anne windows in the left wing of the building. Since the infirmary was founded—in 1750—it has been so enlarged, improved, and new wards built on to the main structure that, architecturally, it is a conglomeration of odd buildings massed together, and all tarred—metaphorically speaking—with the same brush in the coal dust hue of industrious Newcastle.

Once this building, I mean the 1750 part of it, stood on a sunny slope surrounded with bright foliage, through which the Skinners burn, a tributary of the Tyne, rippled pleasantly. Now the burn is a closed-in sewer, and the foliage has disappeared with the coming of the cattle market and the road of iron. The most conspicuous object on entering the porter's lodge is the Royal Coat of Arms, ablaze in all the orthodox colours, hanging over the principal entrance. This 1750 part of the hospital is, indeed, quaint with its dimly lit corridors, its old oaken doors, and stairway with its gigantic grandfather's clock on the first landing. The old timepiece has stopped ticking years ago.

The ground and first floors of the old part are mostly used by the administrative department. Of course, there is a casualty room on the ground floor, and this is lighted by a Queen Anne window. As I passed the open doorway and looked in, a man's head was being bound up in wool and rags. The yellow light from the window fell on his pale features. Here was a possible picture if the costumes of the figures had been in keeping with the quaint lines and curves of the old window —the corduroys of the patient, the black frock of the surgeon, and the collar and cuffs of the nurse, as our American cousins would say, were not "away back" enough.

The chapel is a very interesting feature in this old building. A rather incongruous privilege this chapel enjoys considering the always sad object for which hospitals exist. It carries a licence to marry. Is this provision made in case the irresistible charms of the matron, sisters, and nurses are too much for any male inhabitant? Surely the equanimity of the surgeons with their stern mission in life is not supposed to be disturbed by the opportunity the old chapel affords.

The library and board-room are two charming old rooms. The former has a stock of books almost as antique as the Chippendale cabinets in which they rest. There is also a fine modern collection. The latter room is wainscoted with old oak, and walled with a veritable collection of old masters. The most conspicuous of these portraits is that of the founder of the institutions, Joseph Butler, Bishop of Durham, painted by Taylor in 1750. On the actual canvas, beside the presentment of the good Bishop, is written in white paint—"Annually subscribed £100 to the hospital. He gave £50 towards the new buildings, and by his will left £1,500 to this charity; died at Bath, June 27th, 1752." Above the door opening into the garden is a long rigmarole re Pigg's charity of 1688. There are two portraits in this room, which are quite valuable works of art—Sir Walker Blackett, painted by Sir Joshua Reynolds, and Algernon, fourth Duke of Northumberland, named the good Duke, painted by Sir Francis Grant, P.R.A. There is also a portrait of the Bishop of Gloucester, 1753, and an engraving of the most famous of them all, Robert Stephenson, Esq., civil engineer. The most certainly novel feature in this administrative quarter of the hospital is the electric light. Of course, the operation theatre is fixed up with the incandescent burners, and this room is one of the latest additions to the hospital.

A brass plate on one of the walls has this inscription: "This operating theatre, together with extensive alterations in several parts of the main building, were completed at the sole expense of Sir W. C. Armstrong, C.B., A.D. 1872." This little theatre is certainly one of the best in the three kingdoms, and probably its operating staff is one of the ablest in the world. I happened to be in the theatre when that eminent North-country surgeon, Mr. Page, was at work. He started on an abdominal section at ten o'clock that morning, and within an hour and a quarter he had successfully finished three abdominal sections and one cancer case of the left breast. The systematic way the whole thing was done, the splendid discipline of the sisters, nurses and assistants was a sight worth seeing in spite of the necessarily painful object for which they were assembled. I believe Mr. Page's best record was seventeen first-class operations in one morning between the hours of nine and the hour after noon. I saw the four cases I had seen operated on later in the day, and they were all doing well, and in one, an ovarian case, the patient seemed quite bright and happy. The most noticeable feature in the surgical department of this hospital is the large number of abdominal operations. This last year there were 113 cases in which the abdominal cavity was opened. The total operations during the year of all kinds have been 1,647, an increase over 1892 of 476. There have been 85 deaths after operations, giving a percentage mortality of 5·19, as against 5·3 in the previous year. Considering the age of the building and the necessary danger arising therefrom, the percentage is indeed a remarkably low one. There are three wards in the old building for children—one for boys, another for babies of both sexes, and another for girls, having thirty-two cots in all. The wards as well as the ordinary surroundings of toys and bright pictures for the young, were decorated with a number of beautiful flowers, which, I ascertained, were the gifts of

The Operation Theatre, Royal Infirmary, Newcastle-on-Tyne.

ladies of the theatrical profession starring the town, who seem to be much devoted to this section of the hospital. A less interesting ward in the old part is a small Magdalene ward containing twelve beds.

Leaving the 1760 wing of the building, we move down the better lighted corridor of the 1800 period, and pass on the right three wards with seven, three, and four beds respectively, in which isolated cases are placed. The two flats above —for the building is in three storeys—have wards similar in size and number of beds. Before entering the lower flat of the 1852 part of the infirmary, which runs at right angles to the 1880 structure, we turn to the left into the Ravensworth ward, erected in 1882, and the best ward in the buildings. It is a lofty, oblong room, partitioned with a short screen running down the centre, with double rows of beds in either section making in all 48 beds. The ward is ventilated with Tobin's tubes, and heated by double open stoves at either end of the screen, and the stove shafts have Boyle's patent air extractors. At one end of the ward are the lavatories and bath-room, cut off from the rest of the ward by a short gallery, through which there is always a fresh current of air.

The Ravensworth wing has the appearance of a temporary building, so lightly is it constructed in comparison with the rest of the hospital. In virtue of the thinness of the roof and walls, it is extremely hot in the summer months. To counteract this there are water sprinklers attached to the roof, which, in hot weather, are turned on for cooling purposes. The Ravensworth is used for surgical cases only. The three wards in the 1852 wing are as spacious as this latter one, *re* the number of beds, but by no means so lofty. They are divided by a wall screen similar to the Ravensworth. The lower flat, the Percy and Victoria wards, are for surgical cases; and the two upper floors, containing the same number of beds, are devoted to the medical. The beds in these wards look excessively comfortable, with neat little Duchesse bed top and side curtains in grey material. The lavatory and bath-rooms, though serving their purpose well, are by no means as good, from a sanitary point of view, as those in the latest ward. The whole hospital will comfortably hold 270 beds, and at a pinch the wards are large enough without much inconvenience to take 290, the extra beds being placed down the centre of the sections. During the last twelve months special departments have been established, including one for diseases of the skin and one for diseases of the throat and ear. The ophthalmic department, which has now been in operation for several years, has treated during the past year 1,175 cases. The out-patient department, laundry, stores, and kitchen are in the basement.

The out-patient department is probably the chief drawback to this admirable institution. The waiting-room is small, badly lighted and ventilated, and excessively gloomy. Considering that nearly 17,000 patients are yearly relieved in this department, an improved or new building is urgently needed. The consulting-rooms, opening on to the waiting-room, are in keeping with the rest of this depressing place. The dispensary is a good one. The laundry and wash-house are excellent in every way. There is one large rotary washer and one wringer worked by steam or gas; 8,000 pieces can be easily washed in a week. As there is more surgical work than medical in the Newcastle Infirmary, 100 towels a day on an average are used in the theatre. The airing-room of the laundry is large and well ventilated, and there are 20 drying racks. The kitchen is spacious, and has one of Benham and Sons patent ranges, which was put in with the rest of the kitchen gear during the great alterations in 1879. The cook turns out 350 dinners daily. In the scullery are three large coppers and a steamer for puddings and potatoes. The larders are walled with tiles, and the dairy is one of the best I have yet seen in the provinces.

The Newcastle Infirmary is the great surgical centre for Northumberland, Durham, and part of Westmoreland and Cumberland. The nurses and general staff number fifty-six. The day nurses sleep on the premises, but the night nurses go to a private house in the vicinity of the hospital. The convalescent home for the infirmary is at Whitley, where there are as many beds as required.

But Newcastle is growing daily, and really wants a much larger infirmary. The accommodation is very scant in the face of the numerous applications, especially for women. Here is a chance for a philanthropic millionaire, or a well-to-do British peer. Since the demise of the good old Duke, whose presentment by Sir Francis Grant is in the board-room, little has been done for the Newcastle Infirmary by that great house which draws its vast income from the north country. The famous emblem of the house of Percy—the straight-tailed Lion—scowls on a pedestal in front of the main entrance of the hospital with a stony indifference to the misery and suffering around him. On looking at this noble beast it struck me that the tail was not quite as orthodoxically horizontal as it might be. I found that the brute had met with an accident, and that the surgeon who had attended him, not so familiar with stone as flesh, had fixed the tail on the wrong way. How did the accident happen? Had some impatient sufferer tried to move the great Northumberland lion by twisting his tail?

## Figures and Tables

**Figure f1:**